# Didactic and Content Quality of Basic Life Support Videos on YouTube: Cross-Sectional Study

**DOI:** 10.2196/69103

**Published:** 2025-11-05

**Authors:** Jasmina Sterz, Yvonne Beaugé, Pia Tueckmantel, Lena Bepler, Armin N Flinspach, Yves Gramlich, René Verboket, Philip Bintaro, Maren Janko, Mairen H Flinspach, Michael Merker, Sven Bepler, Jan T Vollrath, Sebastian H Voß, Miriam Rüsseler

**Affiliations:** 1 Medical Faculty, Institute for Medical Education and Clinical Simulation, Goethe University, Theodor Stern Kai 7, Frankfurt am Main, 60590, Germany, +49 69 6301-84073; 2 Department of Anaesthesiology, University Hospital Frankfurt, Intensive Care Medicine and Pain Therapy, Frankfurt am Main, Germany; 3 Agaplesion Markus Krankenhaus, Frankfurt am Main, Germany; 4 Department of Trauma Hand and Reconstructive Surgery, University Hospital Frankfurt, Frankfurt am Main, Germany; 5 Nephrologische Praxis und Dialyse im Klinikum Peine, Peine, Germany; 6 Department of Anaesthesiology, Intensive Care Medicine and Pain Therapy, Sana Clinic Offenbach GmbH, Offenbach/Main, Germany; 7 Department for Children and Adolescents, Division for Stem Cell Transplantation, Immunology and Intensive Care Medicine, University Hospital Frankfurt, Frankfurt am Main, Germany; 8 Department of Anaesthesiology, BG Unfallklinik Frankfurt am Main, Frankfurt am Main, Germany; 9 VOSS – doctor‘s office, Aschaffenburg, Germany

**Keywords:** cardiopulmonary resuscitation, CPR, basic life support, YouTube, quality assessment, didactic quality, content quality, medical education

## Abstract

**Background:**

Cardiopulmonary resuscitation (CPR) is vital for improving patient outcomes in medical emergencies. Both laypersons and health care professionals often seek guidance on performing CPR. In today’s digital age, many turn to easily accessible platforms such as YouTube for practical skills.

**Objective:**

This study evaluates the didactic and content quality of CPR videos on YouTube using comprehensive checklists and investigates the association between the assigned quality scores and type of publisher, view count, and video rankings.

**Methods:**

Videos were included based on defined search terms and exclusion criteria. Two emergency physicians rated each video independently using validated checklists concerning content and didactic quality. Linear regression analysis was performed to assess the relationships between video quality scores and view counts, as well as video rankings.

**Results:**

Of the 250 videos identified, 74 (29.6%) met the inclusion criteria. On the content checklist, videos scored an average of 56.5% (SD 19.2%), and on the didactic checklist, they scored 66.6% (SD 14.3%); none achieved the maximum score. Videos from official medical institutions scored significantly higher in content quality compared to nonofficial sources (*P*=.04). Video quality scores were not associated with video rankings or view counts.

**Conclusions:**

The study highlights substantial variability in the didactic and content quality of CPR-related videos on YouTube. For medical educators, this underlines the need to curate and recommend reliable online resources or to develop new high-quality content aligned with established checklists. For the general public, the findings caution against relying on popularity metrics as indicators of accuracy and emphasize the importance of guidance from trusted institutions.

## Introduction

Ischemic heart disease, the leading global cause of death, presents a substantial challenge to health care systems worldwide [1,2]. In Europe, the incidence of out-of-hospital cardiac arrest ranges from 67 to 179 cases per 100,000 inhabitants [3]. Alarmingly, data from the German resuscitation register show that only 11.1% of individuals who experience out-of-hospital cardiac arrest survive to hospital discharge [4].

Prompt and proficient cardiopulmonary resuscitation (CPR) substantially improves patient outcomes [5-8]. However, gaps in knowledge and performance persist among both laypersons and health care professionals, particularly in essential aspects such as compression depth, hand placement, and compression rate [9-12]. These deficits are further compounded by delays between arrest recognition, CPR initiation, and the arrival of professional medical support [4].

To address these challenges, widespread CPR education is essential, not only in formal training environments but also through accessible, scalable resources that support independent learning. In recent years, especially during the COVID-19 pandemic, online video platforms such as YouTube have become increasingly popular sources for acquiring or enhancing practical medical skills, including CPR [13]. While this accessibility offers great potential, concerns remain regarding the quality, accuracy, and didactic effectiveness of CPR videos freely available online [14,15].

Multiple studies have identified quality deficiencies in YouTube videos on resuscitation [16-27]. For instance, Yaylaci et al [28] reported that only 11.5% of resuscitation videos correctly demonstrated the necessary steps. Similarly, Elicabuk et al [29] reported that 75.3% of Turkish-language CPR videos failed to adhere to current guidelines. However, most previous evaluations relied on limited or nonvalidated assessment criteria [30,31]. This highlights the central problem: learners are widely exposed to CPR videos of uncertain quality but lack reliable indicators of which resources provide accurate and pedagogically sound instruction.

To address this gap, 2 validated checklists were developed: the didactic quality checklist by Rüsseler et al [32] and the content quality checklist by Sterz et al [33], the latter aligned with American Heart Association guidelines [31]. These instruments offer a structured way to evaluate both the educational effectiveness and the technical accuracy of resuscitation videos [32,33].

This study is the first to apply these validated checklists to a sample of English- and German-language YouTube CPR videos, while also examining associations between video quality and publisher type, view count, and search ranking. By integrating didactic and content analysis with visibility metrics, it provides a systematic evaluation of freely available CPR videos and highlights implications both for educators and for the general public.

## Methods

### Study Design and Setting

A retrospective, cross-sectional study design was used. The dataset includes videos uploaded between February 2010 and October 2018 and reflects the state of available content during that period. The video assessment is designed to address a broad target audience, including medical laypersons, professionals, and students.

The evaluation focuses on didactic quality, defined as the appropriateness of educational styles used in the videos, and content quality, which encompasses the procedural correctness of depicted resuscitation techniques and the accuracy of explanations regarding the resuscitation algorithm. Differences in assigned didactic and content quality scores are examined between videos published by official medical institutions or organizations and those from nonofficial sources. In addition, the analysis investigates potential associations between video quality scores and platform metrics such as view counts and search rankings, providing insights into the relationship between video quality and audience engagement within that historical context.

### Video Selection Process

The video selection followed a multistage procedure. In October 2018, a search was conducted on YouTube using a German IP address and 10 relevant keywords in both English and German, including chest compression (“Thoraxkompression” and “Herzdruckmassage”), CPR, basic life support, cardiac arrest first aid (“Herzstillstand erste Hilfe”), heart first aid (“Herz erste Hilfe”), and resuscitation (“Wiederbelebung”). These terms were chosen to ensure thematic relevance and to capture common linguistic variations used by both laypersons and professionals.

To reflect typical user behavior, where users predominantly engage with results from the first search page, only the first 25 videos returned for each term were screened. Videos uploaded between 2010 and 2018 were included, acknowledging the ongoing visibility and ranking of older content within YouTube’s algorithm. For each video, metadata such as view count, number of likes and dislikes (noting that public dislike counts were removed in 2021), uploader identity, upload date, video duration, and channel subscriber count were documented.

### Inclusion and Exclusion Criteria

Videos were included regardless of their explicitly stated target audience, as this information is often missing from video titles or descriptions. Instructional videos were also considered, even if not explicitly labeled as such.

Exclusion criteria comprised videos related to pediatric or animal resuscitation, content in languages other than English or German, videos lacking visual content (eg, audio only), demonstrations of mechanical compression devices, real-life or intraoperative resuscitation footage, and content focusing solely on cardiac arrest prevention without instructional guidance. Additionally, parody, satire, comedy, entertainment content, and promotional material for CPR training courses were excluded. Duplicate videos were removed based on identical URLs. An overview of the selection process is provided in the Results section.

### Reviewer Selection

In total, 16 experienced emergency physicians participated as reviewers in the evaluation process. These individuals were selected based on their extensive expertise and experience in the medical field. The panel included 10 (63%) male physicians and 6 (37%) female physicians, representing a slightly skewed gender distribution toward male physicians. The panel consisted of emergency physicians specialized in various fields, including pediatrics, internal medicine, oral and maxillofacial surgery, trauma care, and anesthesiology. All reviewers actively practice within emergency department settings. Addition, emergency physicians from different German cities, both from university-affiliated and nonuniversity settings, were intentionally included to align with training regulations. This ensures that individuals responsible for resuscitation have relevant experiences and knowledge, regardless of their practice location or institutional affiliation.

### Didactic and Content Checklists

Two separate reviewers assessed each video using 2 distinct checklists: the didactic checklist (Checklist 1), developed by Rüsseler et al [32], and the content checklist (Checklist 2), developed by Sterz et al [33]. The mean of both ratings was used for subsequent analyses. In the event of markedly divergent ratings, cases would have been reexamined to ensure plausibility.

### Scoring Methodology

The didactic checklist consisted of 21 items, each rated on a 5-point Likert scale (1=strongly disagree to 5=strongly agree), yielding a maximum possible score of 105 points. Didactic quality was evaluated based on various checklist aspects, including the title; learning goals; content and technique; content; text, graphics, and images; logical sequencing; aspects of hygiene; target audience; video length; readability of text, graphics, and images displayed; camera perspective; and the quality of auditory and visual elements.

The content checklist comprised 25 items across 4 domains: initial measures (7 items), chest compressions (8 items), automated external defibrillator (AED) use (6 items), and ventilation (4 items). Each item was scored on a 3-point scale (0=not mentioned, 1=incomplete or incorrect, and 2=correct), resulting in a maximum possible score of 50 points.

### Applicability and Score Normalization

Not all checklist sections were applicable to every video. For example, many videos intended for layperson training did not include AED use or ventilation. In such cases, nonapplicable items were excluded entirely from both the maximum possible points and the score. Each video was thus assessed only on relevant items.

To allow for comparison across videos with varying scopes, scores were normalized. The achieved score was divided by the maximum applicable score for that specific video and multiplied by 100 to yield a percentage. For instance, a layperson-focused video with 20 applicable items (maximum score=40) that achieved all 40 points would receive 100%, just like a more comprehensive video with 25 applicable items and a maximum score of 50 that also achieved full marks.

### Data Analysis

The data were recorded using Microsoft Excel. Statistical analysis was conducted using Minitab (Minitab Inc) and SPSS (version 26; IBM Corp). Videos were categorized by language, duration, view count, and publisher type (official vs nonofficial sources). Content and didactic quality scores were analyzed descriptively. For continuous variables, results are reported as means and SDs; categorical variables are presented as frequencies and percentages. Missing values were excluded. The 5 highest-rated, 5 lowest-rated, and 5 most-watched videos were identified to illustrate score extremes.

For group comparisons, Student *t* tests were applied to examine differences in quality scores (content and didactic) between official and nonofficial publishers. Assumptions of independence, normality, and equality of variances were considered: independence was given by design, normality was assessed using the Shapiro-Wilk and Kolmogorov-Smirnov tests, and equality of variances was evaluated with the Levene test. Cohen *d* was calculated as a measure of effect size.

Associations between video metrics (view count, YouTube ranking, and publisher type) and quality scores were examined using regression analyses. Linear regression was selected because the outcome variables (checklist scores) were continuous. Model assumptions were systematically evaluated using the diagnostic output from Minitab: scatterplots of predictors against outcomes (linearity), residuals-versus-fitted plots (homoscedasticity), and Q-Q plots of residuals (normality). Outliers and influential observations were identified through scatterplots and regression diagnostics, and sensitivity analyses were conducted with and without these data points to assess robustness. *R*^2^ values were reported as measures of explained variance (effect size). Regression analyses were conducted in Minitab using a 95% confidence level. A *P* value of .05 or less was considered indicative of statistical significance.

### Ethical Considerations

The Ethics Committee of the Faculty of Medicine, Goethe University Frankfurt, confirmed that no formal ethics approval was required for this type of educational research, as it does not constitute a biomedical research project in the sense of the Declaration of Helsinki and the requirements of §15 of the Professional Code of Conduct for Physicians in Hesse. The study was conducted in accordance with the Declaration of Helsinki.

## Results

### Overview

**Figure 1.**
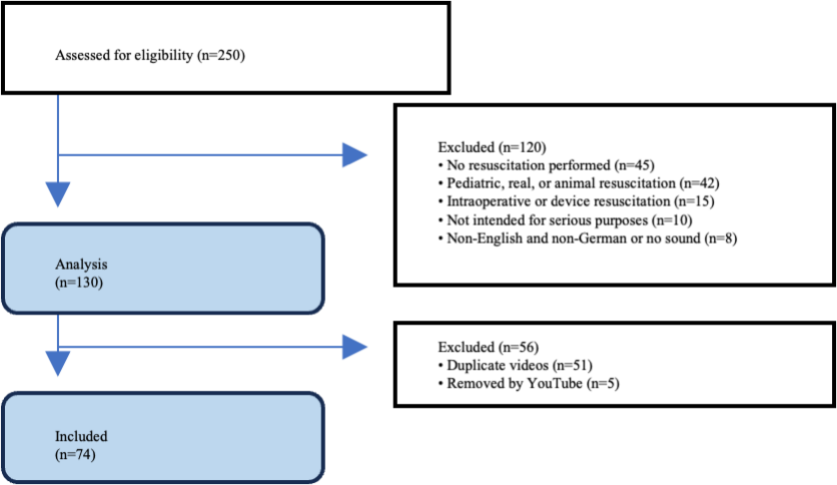
Flowchart of YouTube video selection (retrospective cross-sectional study; YouTube videos accessed via German IP: 2010‐2018; N=74). A total of 250 YouTube videos were screened. After applying predefined exclusion criteria and removing duplicates, 74 unique videos were included in the final analysis.

Initially, a total of 250 videos were identified based on the search terms. Among these, 120 videos were excluded due to meeting at least one exclusion criterion. Subsequently, 51 duplicate videos were identified and excluded, while an additional 5 videos were removed from the online platform before the rating process. This led to the final analysis comprising 74 videos (Figure 1).

### Video Characteristics

The timeline of video uploads spanned from February 1, 2010, to October 26, 2018. Among the included videos, 38 were in English, whereas 36 were in German. Video durations ranged from 13 seconds to 1 hour and 49 minutes. View counts for these videos varied considerably, ranging from 59 to 3,202,821 views. Notably, English-language videos generally garnered more views than their German counterparts, as of November 30, 2018. Table 1 provides a summary of the key characteristics of the videos.

**Table 1.** Key characteristics of the YouTube cardiopulmonary resuscitation videos (retrospective cross-sectional study; YouTube videos accessed via German IP: February 1, 2010-October 26, 2018; N=74).

Characteristics		Videos
Language, n (%)		
English		36 (49)
German		38 (51)
View count, mean (range; SD)		126,002 (59-3,202,821; 395,913)
≤60, n (%)		1 (1)
61‐60,000, n (%)		50 (68)
60,001‐120,000, n (%)		7 (10)
120,001‐300,000, n (%)		11 (15)
300,001‐3,000,000, n (%)		4 (5)
>3,000,000, n (%)		1 (1)
Duration (minutes:seconds), mean (range)		7:05 (0:13-60:49)
≤1:00, n (%)		1 (1)
1:01-5:00, n (%)		45 (61)
5:01-10:00, n (%)		17 (23)
10:01-20:00, n (%)		6 (8)
>20:00, n (%)		5 (7)
Publisher, n (%)		
Official medical institution		13 (18)
Other sources		61 (82)

### Didactic Quality Evaluation

None of the videos achieved the maximum score of 100% on the didactic-related checklist. The average score on the didactic checklist was 66.6% (SD 14.3%) with one video achieving the highest possible score of 94.7%. Conversely, the lowest didactic quality score observed was 33.6% (Table 2). The didactic quality evaluation revealed significant disparities across single items, with higher ratings for visual (62.3% strongly agree) and audio (52.3% strongly agree) aspects indicating effective presentation, contrasted starkly by critical deficiencies in hygiene (57.7% strongly disagree) and in-depth reading (64.6% strongly disagree), underscoring urgent areas for improvement in the didactic approach. Table 3 presents the top 5 highest-rated videos, the 5 lowest-rated videos, and the 5 most-watched videos, along with their specific didactic quality scores and characteristics.

**Table 2.** Distribution of didactic quality scores (retrospective cross-sectional study; YouTube videos accessed via German IP: 2010‐2018; N=74).

Scores	Videos^a^
≤50.00, n (%)	11 (15)
50.01‐60.00, n (%)	16 (22)
60.01‐70.00, n (%)	14 (19)
70.01‐80.00, n (%)	16 (22)
80.01‐90.00, n (%)	14 (19)
≥90.01, n (%)	3 (4)

a Mean 66.6% (SD 14.4%); range 33.6%‐94.7%.

**Table 3.** Top 5, bottom 5, and most-viewed YouTube videos by didactic quality score (retrospective cross-sectional study; YouTube videos accessed via German IP, 2010‐2018; N=74).

Category and rank	Reference	Channel	Didactic score	Content score	Language	Year	Views, n	Duration (h:min:sec)
Top 5 videos by didactic quality score								
1	[34]	DRK Rettungsdienst Mittelhessen	94.7	92.5	German	2018	18,119	0:09:26
2	[35]	SIKANA English	92.6	77.5	English	2016	5240	0:02:55
3	[36]	Arbeitsgemeinschaft für Notfallmedizin	92.2	51.4	German	2015	4911	0:20:16
4	[37]	Das Weltrettungsforum im Namen der Wahrheit	88	55.2	German	2017	59	0:43:17
5	[38]	Saxe Healthcare Communications	86.7	11.9	English	2017	122,466	1:00:49
Bottom 5 videos by Didactic quality score								
1	[39]	nordbayern.de	33.6	48.1	German	2016	5522	0:00:13
2	[40]	LearnEngg	33.8	61	English	2016	314,273	0:03:25
3	[41]	American Heart Assoc.	38.7	56.3	English	2013	82,275	0:03:32
4	[42]	Conny X	44.3	25.8	German	2013	2406	0:04:19
5	[43]	H1 Fernsehen	45.3	34	German	2013	38,117	0:04:24
5 most-watched videos								
1	[44]	CPRCertified	74.9	82.7	English	2014	3,202,821	0:04:58
2	[45]	Weisbrod Imaging	86.3	93.6	English	2012	1,204,871	0:08:59
3	[46]	ProCPR	78.7	64.6	English	2011	600,031	0:06:30
4	[47]	tracy	76.2	59.6	English	2013	378,706	0:01:44
5	[40]	LearnEngg	33.8	61	English	2016	314,273	0:03:25

### Content Quality Evaluation

#### Overview

None of the videos achieved the maximum score of 100% on the content-related checklist, with an average total score of 56.5%, about 10% less than the average didactic score. This indicates that videos demonstrated slightly higher didactic than content quality. The highest content checklist score observed for a video was 95.2%, while the lowest recorded score was 6.7% (Table 4). Upon detailed analysis of specific procedures within CPR instructional videos, it is observed that “Set pain stimulus” is mentioned in only 14.9% of the videos, whereas “Shortest possible hands-off time” is mentioned in 22.3%, and “Allowing complete chest recoil” is mentioned in only 29.7% of the videos. Conversely, “Make an emergency call,” “Frequency of 100‐120/min,” and “Correct pressure point” were correct in 70.3%, 68.9%, and 66.9% of videos, respectively. Table 5 includes a selection of the top 5 highest-rated and lowest-rated videos, along with the 5 most watched videos and their related content quality score.

**Table 4.** Distribution of content quality scores (retrospective cross-sectional study; YouTube videos accessed via German IP: 2010‐2018; N=74).

Score range	Videos^a^
<10.00, n (%)	1 (1)
10.01‐30.00, n (%)	5 (7)
30.01‐50.00, n (%)	20 (27)
50.01‐70.00, n (%)	34 (46)
70.01‐90.00, n (%)	9 (12)
≥90.01, n (%)	5 (7)

a Mean 56.5% (SD 19.3%); range 6.7%‐95.2%.

**Table 5.** Top 5, bottom 5, and most-viewed YouTube videos by content quality score in percent (retrospective cross-sectional study; YouTube videos accessed via German IP: 2010‐2018; N=74).

Category and rank	Video reference	Channel	Didactic score	Content score	Language	Year	Views, n	Duration (h:min:sec)
Top 5 videos by content quality score								
1	[48]	LearningInn	73.6	95.2	English	2013	151,401	00:04:53
2	[49]	heartcom UG	86.3	93.6	German	2015	52,113	00:06:25
3	[34]	DRK Rettungsdienst Mittelhessen	94.7	92.5	German	2018	18,119	00:09:26
4	[50]	ercEuroResusCouncil	77.1	92.4	English	2013	42,339	00:13:31
5	[51]	Thieme	80.5	90.5	German	2015	52,937	00:03:32
Bottom 5 videos by content quality score								
1	[52]	Dr. Heart	59.5	6.7	German	2016	4330	00:02:29
2	[38]	Saxe Healthcare Communications	86.7	11.9	English	2017	122,466	01:00:49
3	[53]	Liverpool John Moores University	56.0	19.6	English	2013	7407	00:01:19
4	[54]	CPR Council	54.3	22.4	English	2015	20,391	00:03:24
5	[55]	SAT.1 Regional	52.1	24.3	German	2015	265	00:02:05
5 most watched videos								
1	[44]	CPRCertified	74.9	82.7	English	2014	3,202,821	00:04:58
2	[45]	Weisbrod Imaging	86.3	93.6	English	2012	1,204,871	00:08:59
3	[46]	ProCPR	78.7	64.6	English	2011	600,031	00:06:30
4	[47]	tracy	76.2	59.6	English	2013	378,706	00:01:44
5	[40]	LearnEngg	33.8	61.0	English	2016	314,273	00:03:25

#### Differences in Quality Score by Publisher Type

Among the 74 videos analyzed, 13 (18%) were published by official medical institutions or organizations, while the remaining 61 (82%) originated from nonofficial sources. Videos published by official medical institutions achieved a content score of 67.5% (SD 20.4%) compared to those from nonofficial sources (mean 54.1%, SD 18.4%). This difference was statistically significant (*t*_16_=−2.18,* P*=.04; 95% CI –26.4% to −0.4%). The effect size was moderate (Cohen *d*=0.71). For didactic quality, official videos scored slightly higher on average (mean 68.9%, SD 15.5%) than nonofficial videos (mean 66.1%, SD 14.3%), but this difference was not statistically significant (*t*_14_=−0.23, *P*=.82; 95% CI −12.7% to 7.1%). The effect size was small (Cohen *d*=0.19).

#### Regression Analysis: Views, YouTube Ranking, and Quality Score

Regression analyses showed that videos with more views appeared to have slightly higher content quality scores (β=.2, *P*=.02, *R*^2^=7.2%). However, this association was influenced by 3 outlier videos with view counts of 600,031; 1,204,871; and 3,202,821. After excluding these outliers, the association was no longer significant (β=.2, *P*=.08, *R*^2^=4.3%). No association was found between view count and didactic quality (β=.2, *P*=.25, *R*^2^=1.9%). Similarly, YouTube ranking was not associated with either content quality (β=.2, *P*=.59, *R*^2^=0.4%) or didactic quality (β=.2, *P*=.08, *R*^2^=4.3%).

## Discussion

### Deficiencies in Didactic and Content Quality of YouTube CPR Videos

This retrospective analysis of CPR-related YouTube videos revealed notable deficiencies in both didactic structure and content accuracy. The average didactic score was 66.6% (SD 14.3%), while the average content score was even lower at 56.5% (SD 19.2%). Importantly, many of the most viewed videos contained inaccurate information or omitted essential steps of resuscitation, such as correct compression technique or emergency call initiation. These results highlight a critical gap in the educational value of widely accessed CPR content and underscore the need for improved quality control in this domain.

### Popularity Versus Quality: The Algorithmic Mismatch

Among the educational videos examined, it became evident that popularity, as measured by view counts, did not consistently align with the quality of didactic instruction or content accuracy. For example, the video with the highest didactic score had only 5200 views, while the video with the highest content score had 151,401 views, neither of which was among the most viewed videos overall. These observations raise questions about the role of algorithms in promoting content to a broader audience, potentially exposing viewers to inadequate or misleading information. Regression analyses confirmed this mismatch: although some associations between popularity and quality reached statistical significance, all models yielded very low *R*^2^ values (<10%), indicating that views and rankings explain little of the variation in video quality and are therefore not reliable indicators of educational accuracy. Better curation and dissemination of accurate, high-quality resuscitation material is essential to ensure that the public has access to reliable information in this crucial field.

### Incomplete Demonstration of Critical CPR Components

The findings align with previous studies by Katipoglu et al [15] and Ferhatoglu and Kudsioglu [14], which also analyzed CPR videos on YouTube and emphasized their poor quality. However, this study differs in its approach, as the checklists encompass a wider range of resuscitation aspects, including the often-overlooked complete chest recoil, revealing significant variations in video quality. While some critical actions, such as making an emergency call, were generally performed correctly, others, such as ensuring complete chest recoil, were frequently inadequately depicted or omitted. Favorable outcomes and high-quality CPR depend on the correct execution of all actions [56]. For instance, even with adequate chest compression depth and frequency, a positive outcome becomes less likely in the absence of chest recoil, which eliminates the diastolic filling phase [57]. In addition, only 22.3% of the videos adequately explained the shortest possible hands-off time, despite its pivotal role in maintaining continuous chest compression, a critical factor for a favorable outcome [58,59].

### Instructional Gaps and the Concept of Conscious Competence

These gaps in video content may be attributed to the concept of “conscious competence” in teaching. Experienced educators often possess unconscious competence [60], automatically performing numerous details correctly without being able to explicitly articulate them. Creators of medical educational videos may also internalize critical aspects, such as chest recoil and minimal hands-off time, treating them as self-evident. To address this challenge, the study used checklists that were developed and validated by medical education experts to meticulously assess didactic and content-related aspects in detail [32,33].

Interestingly, when considering valid outliers, results showed a statistically significant association between view count and content quality score. However, after removing the outliers, no statistically significant association existed between video ranking, view counts, and assigned didactic or content quality scores. This implies again that viewers should exercise caution when relying solely on top-ranked videos to acquire practical medical skills, as these videos may not consistently offer the most accurate or comprehensive information. The recent removal of the dislike button on YouTube further complicates viewers’ ability to accurately assess video quality.

While videos produced by official medical organizations received higher-quality ratings compared to nonofficial sources, it is vital to acknowledge that their videos still achieved an average content checklist score of only 67.5% (SD 20.4%). Therefore, while they may provide valuable insights, they should be approached with discernment rather than being unquestionably recommended for medical education purposes.

### Comparison With Previous Work

The findings echo those of previous research on resuscitation videos and extend to studies on the quality of other instructional videos across a range of medical topics found on YouTube [22,24,28]. For example, Yoo et al [27] in 2020 found no difference in the quality of videos related to knee examinations from professional and nonprofessional organizations. Similarly, Flinspach et al [23] demonstrated a lack of association between the content parameters endorsed by YouTube and the overall quality of videos pertaining to epidural catheterization in obstetrics.

### Implications for Future Practice and Educational Strategy

This study highlights the urgent need for more rigorous standards in the development and dissemination of medical educational videos. Validated checklists, such as those used in this analysis, offer a practical and evidence-based framework for both guiding content creation and evaluating instructional quality. Their integration into the production process can help ensure that videos are pedagogically sound, clinically accurate, and aligned with defined learning objectives. In light of the tendency among experienced practitioners to omit critical explanatory detail due to unconscious competence, content creators, particularly those without formal training in medical education, may benefit from targeted support in didactic design and instructional clarity. Moreover, given that video popularity does not reliably reflect content quality, professional organizations and academic institutions have a responsibility to curate, recommend, or endorse high-quality resources. Such measures are essential to help learners navigate an oversaturated digital landscape and access trustworthy materials.

### Study Limitations

This study has limitations that warrant discussion. First, the retrospective design and focus on videos from 2010 to 2018 narrows the applicability of findings to the current YouTube landscape. However, many of these older videos remain widely accessible and are frequently viewed, and the core CPR principles remain, supporting the continued relevance of the dataset. Second, the study focused solely on freely available content from YouTube, justified by its widespread use as a source of resuscitation information [61]. This choice, while providing a comprehensive dataset, excludes potential insights from other platforms or sources. Additionally, the study included videos without explicit classification as instructional, recognizing that viewers often prioritize popularity metrics over instructional labels. However, this approach introduces variability in viewer interpretation. The restriction to English- and German-language videos reduces the transferability of findings to other linguistic and cultural contexts. Furthermore, reliance on YouTube’s search and ranking system, combined with the decision to analyze only the first 25 results per term to reflect typical user behavior, may have introduced a visibility bias by favoring popularity over educational merit and potentially omitting higher-quality videos less prominently ranked. Interrater reliability was not formally calculated; however, the use of independent dual ratings and validated checklists provided safeguards against individual reviewer bias. Finally, the variation in the applicability of checklist items across different videos, especially regarding aspects related to AED and ventilation, represents an inherent limitation. Given the hands-only approach recommended for laypersons, certain video segments did not pertain to these specific categories, leading to uneven scoring across checklist items. To address this, scores were normalized by excluding nonapplicable items from the total possible points, allowing for a more accurate and comparable assessment.

### Future Research

Future research should build on these findings in 2 ways. First, newer YouTube videos should be analyzed to determine whether content and didactic quality have improved in response to evolving CPR guidelines and changes in the platform’s algorithms. This would provide an updated picture of the educational value currently available to learners. Second, research should move beyond descriptive analyses to test practical interventions aimed at improving video quality and visibility. Examples include integrating validated checklists into the production process, evaluating whether institutional peer review or endorsement increases viewer trust, and examining whether algorithmic adjustments can direct users toward higher-quality content. Together, these approaches would not only monitor the current state of online CPR education but also help identify strategies to actively enhance its reliability and reach.

### Conclusions

This research reveals deficiencies in both the didactical quality and content accuracy of the CPR-related videos available on YouTube. Despite the potential for these videos to disseminate life-saving knowledge, many of them failed to meet fundamental criteria for effective CPR guidance. This educational gap is particularly worrisome, given the crucial roles that laypeople, health care professionals, and medical students play in emergencies. In the rapidly evolving landscape of online education, it is imperative to prioritize the widespread availability of high-quality, accurate, and accessible instructional materials, especially in critical domains like CPR.

Moving forward, medical educators, content creators, and professional organizations should take an active role in ensuring that online CPR education adheres to pedagogical and scientific standards. This may include the broader adoption of validated quality checklists, which inherently support structured video creation processes, as well as institutional endorsement of high-quality content. These measures are essential to empower viewers to make informed decisions about the accuracy of online content and, most importantly, to strengthen CPR training effectiveness and improve patient survival in real emergencies [62].

## Supplementary material

10.2196/69103 Checklist 1 Didactic quality checklist.

10.2196/69103 Checklist 2 Content quality checklist.

## Abbreviations

AED: automated external defibrillator
CPR: cardiopulmonary resuscitation
