# Supplementary material for: Didactic and Content Quality of Basic Life Support Videos on YouTube: Cross-Sectional Study
Source: JMIR Form Res. 2025 Nov 5;9:e69103. doi: 10.2196/69103 (PMC12588586; doi:10.2196/69103)
Supplement: Checklist 1 [file formative-v9-e69103-s001.docx]

**Didactic quality checklist**

**Focus on didactics**

|  | 5 | 4 | 3 | 2 | 1 | 0 |
| --- | --- | --- | --- | --- | --- | --- |
| Title | The title fits perfectly with the content and learning objectives shown. |  | Additional content is shown that is not mentioned in the title.  Or rather, lacks individual content to live up to the title. |  | The content shown does not match the title at all. |  |
| Learning objectives | The learning objectives are explicitly stated in detail at the beginning of the video. |  | The learning objectives are partly named in the course clearly recognizable for the user. |  | The learning objectives of the video are not stated at any time. Or are only reproducible from the content of the video. |  |
| Content/Technique:  Factual and scientific accuracy | The content/techniques shown are factually and scientifically correct. |  | In principle, factually and scientifically correct presentation, but some essential aspects are missing or are presented too briefly and/or not sufficiently correct. |  | Factually and scientifically serious errors (e.g., errors in anatomical presentation). |  |
| Content/Technique:  Detailed process flows/work steps | The work steps are clearly structured and are presented in sufficient detail and, if necessary, explained with graphics. Important work steps are emphasized. |  | Decisive work steps are presented partly precisely, partly imprecisely. |  | Important work steps are shown imprecisely or not at all. |  |
| Content/Technique:  Order | The contents and sequences of actions are presented logically in a meaningful and logical order. |  | Some of the content and plot sequences make sense and are logical. |  | The contents and the consequences of the actions are not or hardly logically presented. |  |
| Content:  Hygiene aspects | Attention is paid to the correct hygienic execution.  Hygiene aspects are sufficiently explained. |  | Aspects of hygiene are insufficiently explained. Individual errors are found in the hygienic presentation |  | Important aspects of hygiene are not presented and/or are presented incorrectly (e.g., make unsterile) |  |
|  | 5 | 4 | 3 | 2 | 1 | 0 |
| Content:  Self-critical implementation | The video encourages self-critical performance of the skill (e.g., in the skills lab).  Indications, contraindications, risks, difficulties, possibilities of errors and their avoidance are sufficiently explained.  The choice of the required material, requirements for the environment, etc. are sufficiently explained. |  | Risks, difficulties, and failure modes are adequately explained in some cases. |  | The indications and contraindications are insufficiently described.  Failure modes and risks are insufficiently explained. |  |
| Content:  Target group | Learning content of the video is appropriate for the target group in terms of scope of content, level of difficulty, comprehensiveness of explanations and type of explanations.  Prerequisite: Target group is named. |  | The learning content is largely appropriate for the target group, but some content is too difficult/simple for the target group. |  | The learning content shown and to be conveyed does not meet the requirements of the target group (e.g. not understandable for the target group in the form presented). |  |
| Content:  Length | Length is absolutely appropriate. |  | The important/relevant content is presented too short/too long in parts (e.g. introduction/abstract). |  | The important/relevant aspects are clearly presented too short/too long. |  |
| Content:  Explanations | Each step shown is sufficiently explained (sound track, text, with graphics if necessary, ...). |  | Partial explanations are included (soundtrack, text, graphics). |  | There are no/clearly too few explanations. |  |
|  | 5 | 4 | 3 | 2 | 1 | 0 |
| Abbreviations and technical terms | Abbreviations and technical terms used are adequately explained and/or identified. |  | Abbreviations and technical terms used are partially explained. |  | Abbreviations and technical terms used are not explained. | none present |
| Camera settings/perspectives | The camera angles/perspectives are used sensibly and show the complete course of action, no abrupt changes. The choice of perspective allows the procedure to be viewed from the perspective that is important for learning the respective work step. |  | The camera angles are sometimes changed abruptly. Due to the chosen perspective, individual sequences of action are sometimes not sufficiently recognizable. Another camera angle would sometimes be desirable. |  | The camera angles are inadequate, relevant perspectives are not brought, important movements are obscured by the camera perspective. |  |
| Texts/graphics/images:  Insert | Sensible use of graphics and picture elements. Graphics and picture elements clarify and support the important contents and learning objectives. |  | Graphics and picture elements are sometimes used sensibly. |  | Clearly too many/too few  Graphics and image elements.  These are not used appropriately. | none present |
| Texts/graphics/images:  Scope | Texts/graphics/pictures are appropriate to the topic and time frame. |  | Mediocre integration of text/graphics/images in terms of subject matter and time. |  | Use of texts/graphics and image elements is inadequate with regard to the scope of topics and time (clearly too many/too few, too long/short). | none present |
| Texts/graphics/images:  Readability | Font/graphic type, size, color are well recognizable and readable and clear. |  | Font/graphics partly not sufficiently recognizable/readable, e.g. image overlaid by text. |  | Texts/graphics/pictures are unclear, not adequately readable. | none present |
| Texts/graphics/images:  Will fade in long enough to read. | Agree |  | Agree in parts |  | Do not agree | none present |
| Texts/graphics/images:  Insert | Important work steps, problems, possibilities of errors, contraindications will be |  | Partial |  | Barely/insufficiently | none present |
|  | 5 | 4 | 3 | 2 | 1 | 0 |
| Texts/graphics/images:  Insert | sufficiently emphasized by texts/graphics/pictures |  |  |  |  |  |
| Quality:  Auditory elements | Auditory elements have a flawless quality without background noise, hiss, etc. |  | Auditory elements are of sufficient quality for the most part, but background noise (e.g., telephone ringing, hissing). |  | Insufficient quality of auditory elements, e.g. only background noise on the soundtrack. |  |
| Quality:  Visual representation | High quality of visual display, good resolution  e.g. HD quality. |  | The pictures are partly not sharp, partly the image flow is not smooth. |  | Inadequate picture quality in large parts. |  |
| Sources for background information/extensive reading | Sufficient in-depth and further sources will be mentioned. |  | Other sources should be cited. |  | No in-depth and further sources are mentioned or these are clearly outdated. |  |
| Summary/  Take Home Message | Summary and Take Home Message(s) are appropriate to the scope in terms of time and content and reflect the essential learning objectives. |  | Partly |  | Summary and/or  Take Home Messages are inappropriate in terms of timing and content. | none present |
