# Supplementary material for: Didactic and Content Quality of Basic Life Support Videos on YouTube: Cross-Sectional Study
Source: JMIR Form Res. 2025 Nov 5;9:e69103. doi: 10.2196/69103 (PMC12588586; doi:10.2196/69103)
Supplement: Checklist 2 [file formative-v9-e69103-s002.docx]

**Content quality checklist (**Translated from the original German checklist)

**Focus on video content**

| Measure | Not mentioned (0 p.) | False/  Incomplete  (1 P.) | Correct  (2 P.) | Not applicable / already done |
| --- | --- | --- | --- | --- |
| **Initial measures** |  |  |  |  |
| Set verbal stimulus |  |  |  |  |
| Set pain stimulus |  |  |  |  |
| Check airway |  |  |  |  |
| Check breathing |  |  |  |  |
| Make an emergency call |  |  |  |  |
| Undress upper body |  |  |  |  |
| Lay patient flat |  |  |  |  |
| **Chest compression** |  |  |  |  |
| Place patient on a hard surface |  |  |  |  |
| Frequency of 100-120/min |  |  |  |  |
| Correct pressure point |  |  |  |  |
| Compression depth 5 cm |  |  |  |  |
| Allowing complete chest recoil |  |  |  |  |
| Change compressor |  |  |  |  |
| Repeat cycles |  |  |  |  |
| Minimizing interruptions (< 10 sec) |  |  |  |  |
| **AED** |  |  |  |  |
| **Not applicable Sub items omitted** |  | | | |
| 2. priority |  |  |  |  |
| Stick electrodes |  |  |  |  |
| Rhythm analysis without simultaneous compression |  |  |  |  |
| Do not touch patient during shock (2 points only if explicitly announced; 1 point if only performed). |  |  |  |  |
| Immediately after shock, continue chest compressions |  |  |  |  |
| Rhythm control again after 2 min |  |  |  |  |
|  |  |  |  |  |
| **Breathing** |  | | | |
| **Not applicable because lay resuscitation or intubation Sub items omitted** |  | | | |
| 30:2 chest compressions: Ventilation |  |  |  |  |
| Overextend head |  |  |  |  |
| Hold nose and ventilate via mouth/ if mask and bag are available, use them with C-handle |  |  |  |  |
| Pay attention to thorax elevation as feedback  (2 points explicitly announced; 1 point if only performed). |  |  |  |  |
